# Surgical Repair of Bilateral Achilles Tendon Rupture Following Levofloxacin Use

**DOI:** 10.7759/cureus.80011

**Published:** 2025-03-04

**Authors:** Amanda Anderson, Huthayfa Kahf, Matthew Hatter, Naudereh Noori

**Affiliations:** 1 Orthopaedic Surgery, University of California Irvine Medical Center, Orange, USA

**Keywords:** achilles tendinitis, achilles tendon debridement and tenosynovectomy, achilles tendon rupture bilateral, flexor hallucis longus tendon transfer, fluoroquinolones, gastrocnemius lengthening, midsubstance speedbridge, minimally invasive surgery (mis) achilles repair, orthopaedic surgery, percutaneous achilles repair

## Abstract

Fluoroquinolones are widely used in the treatment of common infections such as urinary tract and airway infections. There have been reported adverse effects of causing tendinopathy and tendon ruptures, with limited case reports involving Achilles tendon rupture. Previously documented risk factors include concurrent steroid use as well as systemic diseases such as vascular or rheumatic disease. There is a scarcity in the literature involving acute bilateral Achilles tendon ruptures following fluoroquinolone usage and subsequent surgical treatment options. The purpose of this case report is to present a rare case of a previously healthy and active elderly patient who sustained bilateral Achilles tendon ruptures following levofloxacin usage and underwent bilateral Achilles tendon repairs.

## Introduction

Fluoroquinolones were developed in the 1960s and have been widely used to treat various common infections. Ciprofloxacin and levofloxacin are routinely prescribed for the treatment of urinary tract and airway infections. Despite their utility, reports began documenting tendinopathies and tendon ruptures in the 1980s, which can occur within hours to six months after treatment [1,2]. In 2008, a black box warning from the Food and Drug Administration (FDA) was issued for its drug-induced tendinopathy [3]. Ninety-five percent of reported tendinopathies and ruptures following fluoroquinolone use involve the Achilles tendon [4]. However, fewer than 20 cases exist in the literature of bilateral fluoroquinolone-induced Achilles tendon ruptures, with treatments discussed in these papers consisting of nonoperative management and open Achilles tendon repair [1,2,5]. Risk factors include age greater than 60, male sex, corticosteroid use, renal disease, rheumatic disease, and trauma [6]. The purpose of this case report is to present a rare case of bilateral Achilles tendon rupture following fluoroquinolone usage and to discuss the surgical technique utilized in this patient with challenging poor tendon and soft tissue quality.

## Case presentation

**Figure 1:**
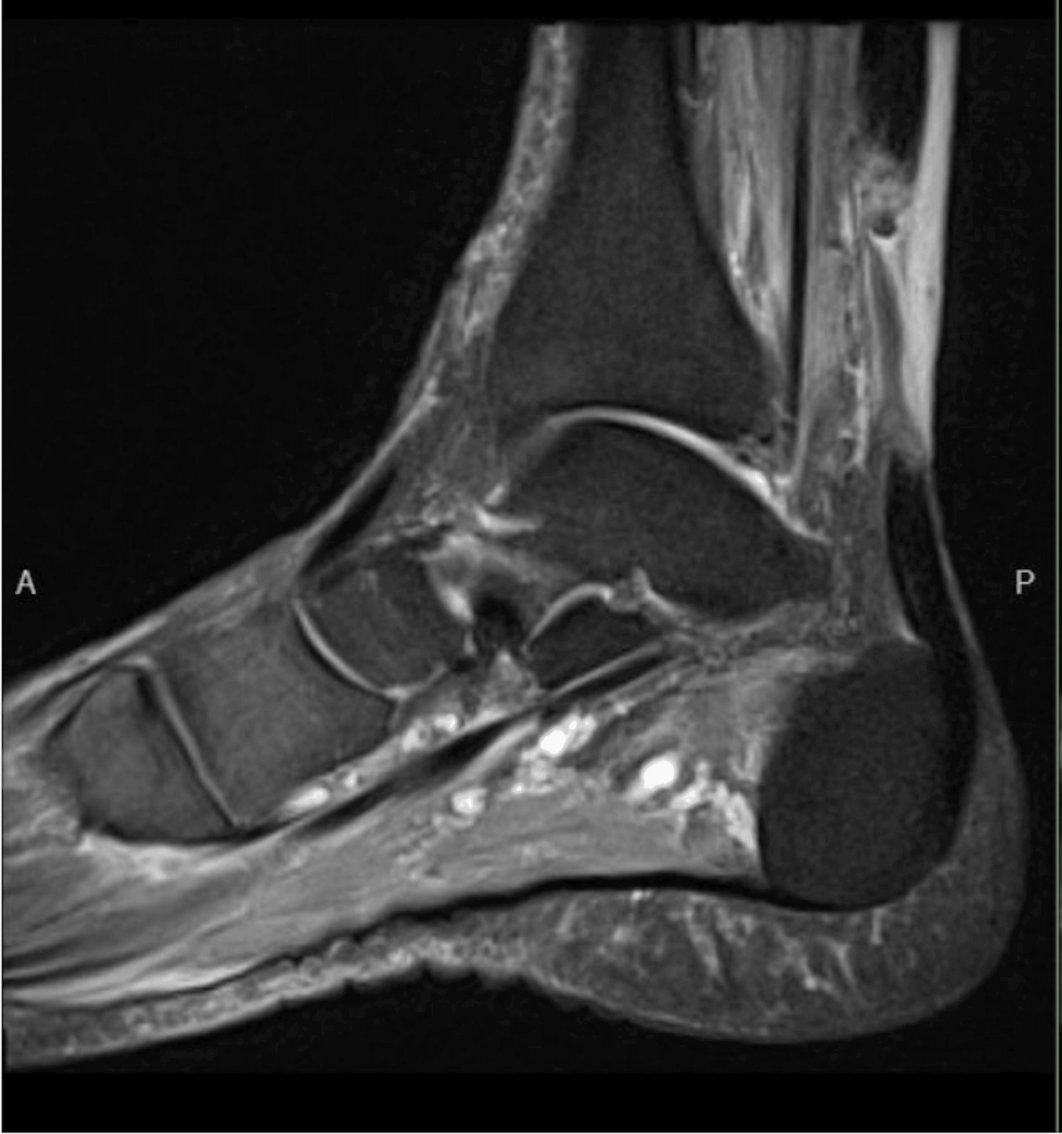
Achilles tendon (left) sagittal PD MRI image PD: proton density; MRI: magnetic resonance imaging

**Figure 2:**
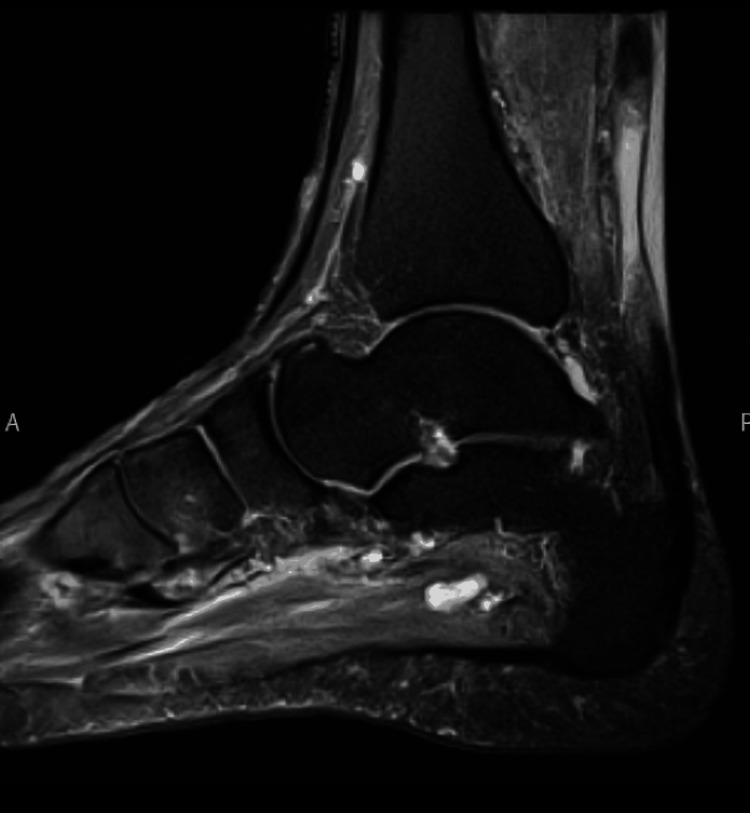
Achilles tendon (right) sagittal PD MRI image PD: proton density; MRI: magnetic resonance imaging

The authors present the case of a 71-year-old otherwise healthy and active male patient who presented with bilateral Achilles tendon ruptures following a course of levofloxacin use for a urinary tract infection. He reported pain and swelling in the bilateral Achilles tendons since starting his antibiotic course. One week after starting levofloxacin, the patient experienced increased bilateral posterior leg pain when he jumped to get a ball playing with his grandchildren. He presented to an urgent care the same day, where he was diagnosed with bilateral Achilles tendon ruptures with a positive Thompson test and palpable midsubstance Achilles tendon defects. He was also noted to have full-thickness tendon tears on ultrasound. Unfortunately, he was not provided with any immobilization and was not placed in plantar flexion. He presented to the orthopedic surgery clinic four weeks later with persistent, significant difficulty walking, having been weight-bearing as tolerated in regular shoes in a neutral position for over a month. Bilateral ankle MRIs were ordered to evaluate tendon quality and assess the distance between tendon edges for preoperative planning due to the subacute presentation (Figures 1, 2). This showed a complete tear of the right distal Achilles tendon in the critical zone, which was approximately 4.3 cm from its calcaneal attachment and with a tendon gap of approximately 4.1 cm (Figure 1). Additionally, a complete tear of the left distal Achilles tendon in the critical zone was observed approximately 3.3 cm from its calcaneal attachment, with approximately 3.4 cm of gap between the tendon edges (Figure 2).

Given the patient's previous baseline health and functional activity level and subacute presentation without immobilization in plantar flexion, it was recommended that he undergo bilateral Achilles tendon repair and reconstruction. Intraoperatively, the Achilles tendons exhibited extremely poor quality, covered in extensive tenosynovitis and fatty infiltration, with an approximately 4 cm gap between the tendon edges.

**Figure 3:**
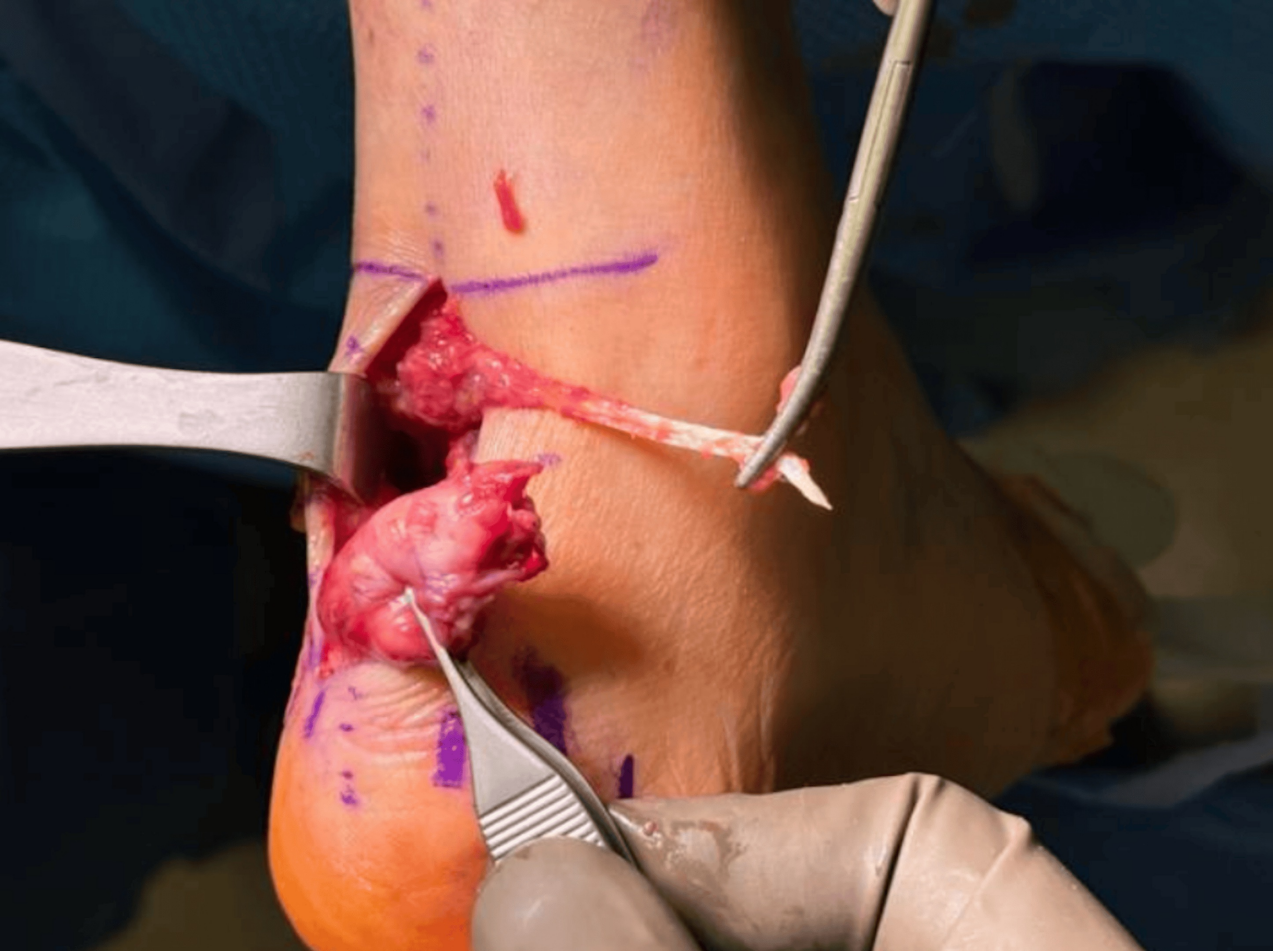
FHL tendon and distal Achilles tendon stump (right) FHL: flexor hallucis longus

The right lower extremity was amenable to repair with the percutaneous Achilles repair system (PARS) of the proximal stump. A Strayer gastrocnemius lengthening was performed to allow end-to-end tendon apposition. A decision was made to perform a flexor hallucis longus (FHL) tendon transfer, given the poor quality of the native Achilles tendon. However, the FHL was also found to be significantly fatty infiltrated and extremely small, with a diameter <4 mm in size (Figure 3).

**Figure 4:**
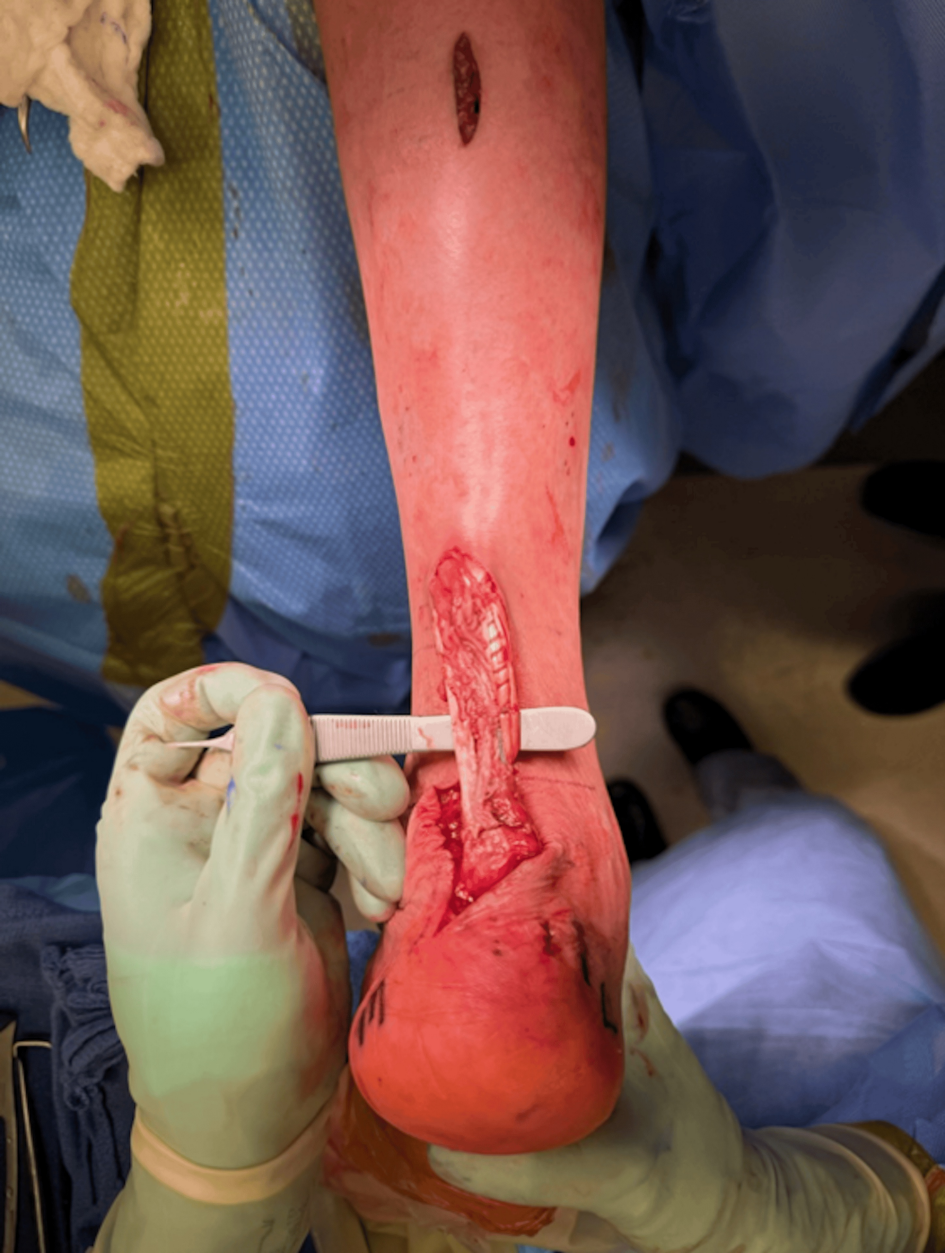
Achilles tendon (right) final repair construct incorporating FHL tendon transfer and semitendinosus allograft augmentation FHL: flexor hallucis longus

The tendon was secured into the calcaneus anterior to the Achilles insertion using a double-loaded mini-FiberTak® anchor (Arthrex, Naples, FL). Achilles repair was completed with a midsubstance Speedbridge construct using 3.9 mm SwiveLock® anchors into the calcaneus (Arthrex, Naples, FL). Given the poor soft tissue quality and diminutive size of the FHL tendon, a decision was made to further augment the tendon and rupture site with semitendinosus tendon allograft. This was incorporated into the proximal and distal stumps using side-to-side tenodesis, and pulvertaft weave proximally with a 1.3 mm suture tape (Figure 4).

For the left lower extremity, the Achilles tendon was similarly of extremely poor quality. This side also required Strayer gastrocnemius lengthening and was also repaired using a PARS-midsubstance Speedbridge technique. Given the diminutive size and quality of the FHL tendon on the right lower extremity, augmentation with semitendinosus allograft was used alone in performing reinforcement of the repair. The final intraoperative clinical exam confirmed improved resting tension and a negative Thompson test bilaterally. Postoperatively, the patient was maintained non-weight-bearing (NWB) for four weeks on the right side and six weeks on the left side. A gentle range of motion was started at two weeks post-op in a controlled ankle movement (CAM) boot, with formal physical therapy starting at six weeks.

At his 10-week follow-up, the patient was doing well, with healed incisions. His pain was controlled, and he was not taking any pain medications. He was able to ambulate without assistance and had begun physical therapy. He had regained the full range of motion of his bilateral ankles.

## Discussion

This case describes the operative management of a rare, subacute presentation of bilateral fluoroquinolone-induced Achilles tendon ruptures in a previously otherwise healthy and active patient. Recent history of fluoroquinolone use has been shown to increase the risk of Achilles tendonitis, particularly in patients over 60 years old and with concurrent oral glucocorticoid use [5]. Observational studies have reported a 2-3% incidence of overall tendon ruptures with a 1.9 to 5.3-fold increased relative risk [7,8]. In a retrospective study of tendon ruptures with seven common antibiotics, levofloxacin was associated with a 120% increased risk of Achilles tendon rupture in 30 days [9], with a peak incidence between 30 and 40 years of age and a second peak between 70 and 80 years of age [4]. The other six antibiotics that were used in this study by Baik et al. included other fluoroquinolones (ciprofloxacin, moxifloxacin) and amoxicillin, amoxicillin-clavulanate, azithromycin, and cephalexin [9].

There is little previous data on similar presentations and surgical treatment options. Prior observational studies have reported an association between fluoroquinolone use and tendon ruptures, possibly due to the upregulation of the metalloproteinase enzyme with collagenase activity, resulting in tendon weakening [10,11]. There have been few reported cases detailing bilateral Achilles tendon ruptures and management in the literature [5,12,13]. Only one previous case report was found on bilateral complete Achilles tendon ruptures on levofloxacin treatment alone without corticosteroids in an elderly patient who was treated nonoperatively [5]. One case report detailed left and right Achilles tendon ruptures occurring two and 10 days, respectively, after starting levofloxacin and corticosteroids, treated with open Achilles repair using side-to-side Orthocord in Krakow fashion [13].

## Conclusions

In our patient, the diffusely poor tendon quality presented technical challenges in attaining a robust repair, requiring reconstruction augmented with FHL and allograft tendon and anchoring into the calcaneus. Whether this is reflective of poor native biology, the subacute nature of the injury, or a result of levofloxacin use, surgeons should be prepared for alternative reconstruction options when treating these patients.
